# Searching for wheat resistance to aphids and wheat bulb fly in the historical Watkins and Gediflux wheat collections

**DOI:** 10.1111/aab.12326

**Published:** 2016-12-12

**Authors:** G.I. Aradottir, J.L. Martin, S.J. Clark, J.A. Pickett, L.E. Smart

**Affiliations:** ^1^ Department of Biological Chemistry and Crop ProtectionRothamsted ResearchHarpendenHertfordshireUK; ^2^ Department of Computational and Systems BiologyRothamsted ResearchHarpendenHertfordshireUK

**Keywords:** Aphid, Delia coarctata, insect, resistance, Rhopalosiphum padi, Sitobion avenae, Triticum aestivum, wheat, wheat bulb fly

## Abstract

Insect pests can reduce wheat yield by direct feeding and transmission of plant viruses. Here we report results from laboratory and field phenotyping studies on a wide range of wheat, including landraces from the Watkins collection deriving from before the green revolution, more modern cultivars from the Gediflux collection (north‐western Europe) and modern UK Elite varieties, for resistance to the bird cherry‐oat aphid, Rhopalosiphum padi (Homoptera: Aphididae) and the English grain aphid, Sitobion avenae (Homoptera: Aphididae). A total of 338 lines were screened for R. padi and 340 lines for S. avenae. Field trials were also conducted on 122 Watkins lines to identify wheat bulb fly, Delia coarctata, preference on these landraces. Considerable variation was shown in insect performance among and within different wheat collections, with reduced susceptibility in a number of varieties, but phenotyping did not identify strong resistance to aphids or wheat bulb fly. Field trials showed within collection differences in aphid performance, with fewer aphids populating lines from the Watkins collection. This differs from development data in laboratory bioassays and suggests that there is a pre‐alighting cue deterring aphid settlement and demonstrates differences in aphid preference and performance on older plants in the field compared with seedlings in the laboratory, highlighting the need for phenotyping for aphid resistance at different plant growth stages. No association was identified between performance of the different insect species on individual varieties, potentially suggesting different nutritional requirements or resistance mechanisms.

## Introduction

Wheat, *Triticum aestivum* L., is the third most important cereal crop in the world and the dominant crop for human consumption in temperate countries. Population growth, coupled with per capita increase in consumption, drives the demand for wheat, which is increasing rapidly (Shewry, 2009; Curtis & Halford, 2014). In context, the global wheat production forecast for 2014 is 718.5 million tonnes globally, with Europe and UK contributions estimated at 236.3 and 16.6 million tonnes, respectively. Wheat price fluctuates, but the average price in the USA in September 2014 was $279 per tonne, making the global production of 2014 worth approximately $200 billion (FAO, 2014; HGCA, 2014). Thus protection of wheat yield is of both economic and social importance and this must include preventing loss of yield to insect pests. Several aphid species are economically important pests of wheat including the bird cherry–oat aphid, *Rhopalosiphum padi* (L.) (Homoptera: Aphididae) and the grain aphid, *Sitobion avenae* F. (Homoptera: Aphididae), which cause damage and yield loss by direct feeding and vectoring plant viruses. Currently, aphid damage to crops is controlled mainly by insecticidal treatments (Tanguy & Dedryver, 2009). However, this is now failing, as a result of the evolution of insecticide resistance, with some species exhibiting resistance to multiple insecticidal classes (Bass *et al.*, 2014). This, coupled with restrictions on the use of some pesticides in Europe, has focused global research efforts to find alternatives to pesticides (Loxdale, 2008; Sparks, 2013).

It is of great importance to the resilience of crops that sufficient genetic diversity is retained and recovered from diverse germplasm in order to respond to pressures of abiotic and biotic stresses, including insect feeding. Reduction in genetic diversity has been reported in modern wheat, compared with wild ancestors, which is thought to be due to population bottlenecks (Doebley *et al.*, 2006), and with the domestication of wheat and varieties becoming adapted to local conditions, giving rise to so‐called landrace cultivars, causing even further reduction in genetic variation (Reif *et al.*, 2005). More recent results from studies on changes in genetic diversity of European wheat varieties over time have suggested that genetic diversity has not in fact decreased, but rather that changes have occurred in alleles present in the germplasm (Huang *et al.*, 2007). Phenotyping of plant collections to search for beneficial plant traits such as resistance to insect pests is a crucial part of developing new germplasm for breeding of elite varieties that will thrive under likely climate change and pesticide constrained conditions. This is increasingly being done on any accessions that could perceivably be useful to crop improvement (Grosskinsky *et al.*, 2015; Rahaman *et al.*, 2015).

The Watkins collection consisted of over 7000 hexaploid bread wheat landraces collected from local markets in 32 countries in the 1930s, with a near global distribution and is curated by the John Innes Centre Germplasm Resource Unit, Norwich. Many have been lost and the current Watkins collection consists of 826 accessions from around the world showing high genetic diversity and thus containing information on the genetic diversity of wheat before the start of modern breeding. A core collection comprising 119 lines, capturing the majority of the genetic diversity, has been defined (Wingen *et al.*, 2014). New alleles and/or genes for leaf rust resistance, stripe rust resistance and root‐lesion nematode resistance have previously been found in the Watkins collection (Dyck, 1994; Bansal *et al.*, 2011; Thompson & Seymour, 2011). The Gediflux collection consists of over of 500 modern wheat cultivars intensively bred and adapted to the narrow geographic region of north‐western Europe, have been sown in major acreage in the years 1945–2000. A study on the genetic diversity of the collection showed no significant change over time (Reeves *et al.*, 2004).

*Rhopalosiphum padi* and *S. avenae* are both important aphid pests of wheat with a near worldwide distribution and have the ability to build up large populations quickly due to asexual reproduction and short life cycles, yield losses of 20–30% due to *R. padi* and 20% by *S. avenae* have been reported (Voss *et al.*, 1997). After severe outbreaks, up to 60% losses were reported in China, where *S. avenae* is the predominant aphid species during the later growth stages of wheat in all wheat producing regions (Li & Peng, 2014). Both species also vector barley yellow dwarf virus (BYDV) which causes leaf yellowing and stunting, resulting in severe yield losses (Leather *et al.*, 1989; Di Pietro *et al.*, 1998).

Wheat bulb fly, *Delia coarctata* Fallén (Diptera: Anthomyiidae) is widespread across Eurasia and North America. It is a major pest of winter wheat in some areas of the UK, where the ground dwelling larvae invade and feed within the tillers of young plants resulting in so‐called ‘deadheart’, that is, the yellowing and death of the central tiller, which can lead to the death of the seedling. Economic damage due to this pest is sporadic and highly dependent on summer rainfall, but yield losses can be as high as 4 t ha^−1^ (Rogers *et al.*, 2015).

The aim of this study was to use phenotyping to search for resistance to the cereal aphids *R. padi* and *S. avenae*, and to *D. coarctata*. Presence of resistance to these wheat pest species is highly desirable for the identification of lines which could be used for the development of insect resistant cultivars and studies into resistance mechanisms.

## Materials and methods

### Aphids and plant material

Cultures of the grain aphid, *S. avenae*, and the bird‐cherry oat aphid, *R. padi*, originating from several individuals collected from the field at Rothamsted Research (Harpenden, Hertfordshire, UK) and which have been kept in culture for over 3 years, were reared under controlled environment conditions (16:8 h L : D 22 ± 1°C) in specially designed ventilated cages in a custom built insectary. *Sitobion avenae* was reared on wheat seedlings of cultivar Tybalt, and *R. padi* was reared on barley, *Hordeum vulgare* L., seedlings of cultivar Saffron. The aphids were overcrowded in order to generate several hundred alate morphs per week for use in bioassays.

In a series of controlled environment bioassays spanning over 3 years, a total of 568 and 569 wheat lines were tested against *R. padi* and *S. avenae*, respectively. The results extracted and reported here relate to the 338 (*R. padi*) and 340 (*S. avenae*) lines included from the Watkins and Gediflux wheat collections. Seeds from the Watkins Core I collection (Wingen *et al.*, 2014) plus other lines were provided by the John Innes Centre Germplasm Resource Unit, Norwich, and seeds from the Gediflux collection from WGIN [Defra (UK) Wheat Genetic Improvement Network]. As all lines could not be tested simultaneously, a commercial variety of *T. aestivum*, Solstice, was used as a control to provide a comparative standard across all bioassays. Overall, *R. padi* was screened on 275 Watkins and 63 Gediflux accessions and *S. avenae* was screened on 277 Watkins and 63 Gediflux accessions.

Seeds were sown into Rothamsted Prescribed Mix 7 days prior to experiments and kept at 20°C (±1°C), 50% humidity, 16:8 h (L : D) regime with daily watering. The mix, supplied by Petersfield Products, Leicestershire, UK, comprises: 75% medium grade (L&P) peat, 12% screened sterilised loam, 3% medium grade vermiculite and 10% grit (5 mm screened, lime free). For each bioassay (see below) 20 seeds of each cultivar and 40 seeds of Solstice were sown in labelled P40 plastic trays and watered from above, to penetrate but not waterlog the compost. Trays were covered with slightly ventilated propagator tops and watered lightly when necessary until the plants germinated, at which point the tops were removed and the seedlings watered as needed. This provided a minimum of 10 evenly sized plants per line plus 20 plants of the cultivar Solstice.

### Aphid resistance phenotyping

Aphid phenotyping bioassays were conducted in identical conditions to those where plants were grown, that is, 20°C (±1°C), 50% humidity and 16:8 h (L : D). Up to 18 wheat lines plus Solstice were tested against one aphid species each week. In each week the lines were allocated according to a two‐block randomised complete block design (RCBD), with an additional replicate of Solstice within each block. Blocks comprised five trays each divided into four rows to give a 5 × 4 array of 20 experimental units, each of which contained five pseudo‐replicate plants of the allocated variety. Alate aphids of 1–2 days after moulting to adult and leaving the plant were collected from the top of culture cages in batches of approximately 100 per tube, two alates were put into each clip cage (MacGillivray & Anderson, 1957) and, when all cages were populated, one cage was put onto the first leaf of each of the 200 plants, which were at growth stage 11–12, that is, with one or two leaves (Tottman & Makepeace, 1979).

Due to differences in rate of development (Greenslade *et al.*, 2016), the timings of bioassays differ by 1 day between the two aphid species, with *S. avenae* alates put onto plants on day 8 and *R. padi* alates on day 9 after sowing. On day 10 after sowing, 24 h (*R. padi*) and 48 h (*S. avenae*) from when the alates were put onto the wheat, they were removed with fine forceps, the number of nymphs produced recorded and the clip cages replaced. On day 14, the numbers of surviving nymphs were recorded and then transferred into pre‐weighed Eppendorf tubes and weighed to obtain average fresh nymph weight (mg).

### Statistical analysis

Three responses were analysed: the number of nymphs produced, the proportion of surviving nymphs and the mean weight of surviving nymphs. Each response was analysed using a linear mixed model (LMM) fitted using restricted maximum likelihood (REML), with variety as the fixed model term and the random model representing the nested design structure of weeks, blocks within weeks, units within blocks and pseudo‐replicate plants within units. Additionally, comparisons were made between and within collections. The nymph numbers and weights were logged (base 10), the former after adding an offset of one to allow for zero counts, to achieve variance homogeneity and normality. The proportion of surviving nymphs was transformed to logits, again incorporating an offset of one to allow for 0 or 100% survival. Data for each aphid species were analysed separately. All analyses were done using GenStat (16th edition).

### Wheat bulb fly field trial

A field trial, consisting of the 119 accessions from the Watkins core 1 collection plus three additional lines and Paragon from the Gediflux collection as a standard, was sown on Lord's Ground Farm, near Swaffham Prior, Cambridgeshire, England, on 25 November 2013 in three blocks in a randomised complete blocking design to account for patchy distribution of *D. coarctata* (Rogers *et al.*, 2015). Plots were 1 × 2 m and separated by 0.5 m unsown surrounds. Watkins lines 1190166 and 1190736 were only replicated twice due to insufficient seed. Sampling was done on 12 March 2014, when all plants from three pseudo‐replicate rows, each of length 0.33 m, were taken from each plot (11–30 plants total per plot). Samples were stored at −20°C until dissection, when number of larvae and tiller damage were recorded. One sample was lost for line 1190496.

### Statistical analysis

The proportions of damaged tillers per plot were analysed using a generalised linear model (binomial error with logit link), allowing for over‐dispersion. The average numbers of larvae per plant per plot were transformed to the square root scale and, due to the presence of the missing samples, analysed using a LMM/REML. Both analyses accounted for the RCBD structure and all analyses were done using GenStat (16th edition).

### Aphid field trial

A field experiment of 172 lines from the Watkins and Gediflux Collections, as well as Elite varieties and a Rye variety, was sown on 22 October 2010 in a five block randomised complete blocking design in Claycroft field at Rothamsted Research Farm, Harpenden, Hertfordshire, UK. Plots were 0.5 × 0.5 m with 0.5 m unsown surrounds. Aphid counts were done on five tillers per plot on 9 June 2011 when plants were at full ear emergence to mid anthesis, (growth stages 59–65), and when all cereal aphid species were present, that is, *R. padi*, *S. avenae* and *Metopolophium dirhodum* (Walker).

### Statistical analysis

Total aphids, as well as number of *S. avenae* and *M. dirhodum* were analysed using analysis of variance (ANOVA). As the total number of *R. padi* were very low at 45 aphids, statistical analysis was not performed for this species. Counts were transformed to logarithms to achieve variance homogeneity and normality, with an offset of one to allow for zero counts.

## Results

### Aphid resistance phenotyping

**Figure 1:**
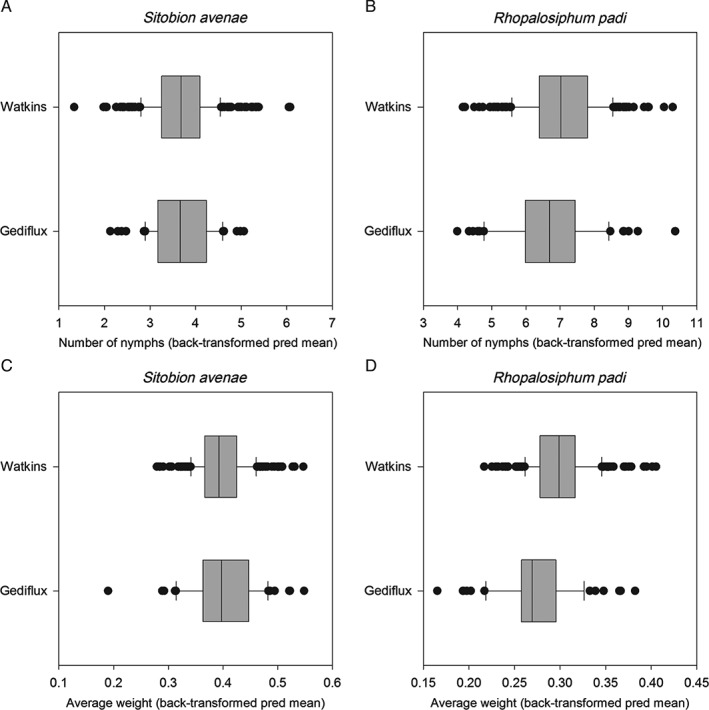
Box plots of back‐transformed predicted means for (a, b) number of nymphs produced and (c, d) average nymph weight, on accessions from the Watkins and Gediflux wheat collections for Sitobion avenae and Rhopalosiphum padi, respectively.

**Figure 2:**
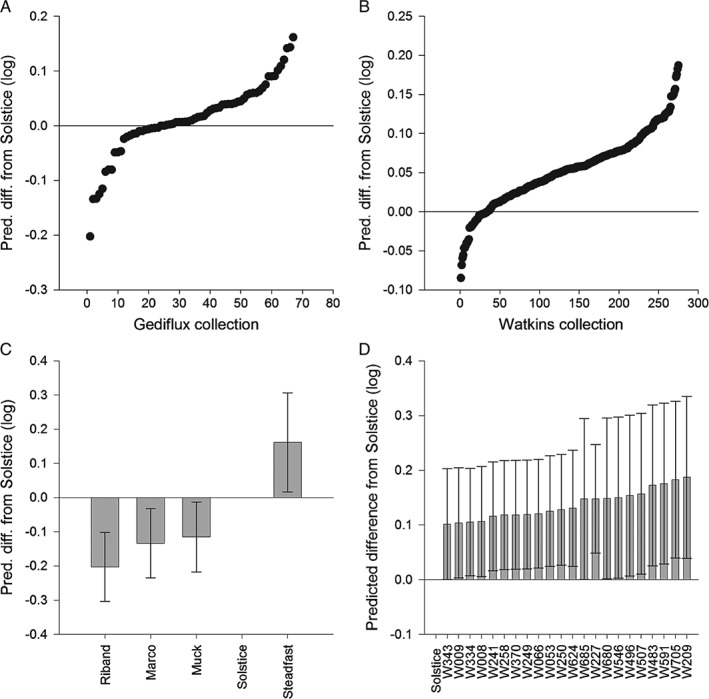
Rhopalosiphum padi predicted differences (log scale) in nymph weight from control (Solstice), for the (a) Gediflux collection, (b) Watkins collection, and lines showing a difference to the control at P < 0.05 for (c) Gediflux collection, and (d) Watkins collection with 95% confidence intervals.

**Figure 3:**
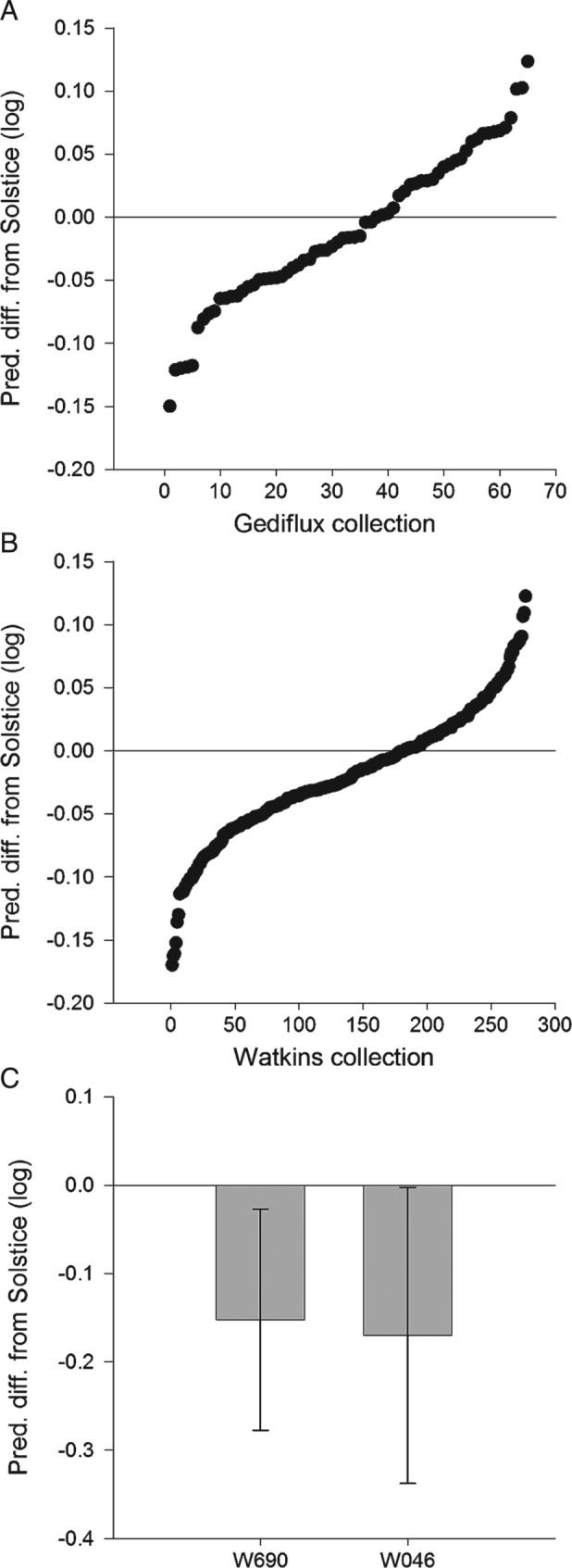
Sitobion avenae predicted differences (log scale) in nymph weight from control (Solstice), for the (a) Gediflux collection, (b) Watkins collection, and (c) lines showing a difference to the control at P < 0.05 which only occurred within the Watkins collection with 95% confidence intervals.

The predicted overall mean responses for the two collections generally did not differ from that of Solstice (Table 1, Fig. 1), but there was evidence for within‐collection phenotypic variation in terms of the achieved average nymph weight for both *R. padi* and *S. avenae* (Table 1, Figs 2 and 3). Out of 63 lines from the Gediflux collection, three were shown to support lower *R. padi* nymph weight and one line on which *R. padi* nymphs were heavier than on Solstice. A considerably larger number of lines were available to be tested from the Watkins collection, where out of 275 accessions, 22 showed increased *R. padi* nymph weight gain compared with Solstice and none showed a lower *R. padi* nymph weight. There were fewer accessions that showed a difference in weight for *S. avenae* nymphs, with just two for each of the Gediflux and Watkins collections, both showing a lower nymph weight gain compared with Solstice. An overall mean weight increase was seen for *R. padi* compared with the Solstice control (Figs 2 and 3). There were no differences in nymph production by *R. padi* on plants from the Watkins compared with the Gediflux collection and *S. avenae* showed a significant reduction in nymph production on just one accession (W053) (data not shown) for the 338 and 340 accessions tested. A within collection difference was observed for the number of *R. padi* nymphs on the Gediflux collection. None of the lines, showing a difference to Solstice, were consistent for aphid species, treatment or variable. The proportion of nymphs that survived after the adults were removed differed only within the Gediflux collection, but not for the other aphid species or collection.

**Table 1:** Predicted mean responses and differences to Solstice of Rhopalosiphum padi and Sitobion avenae on wheat collections (on scale of analysis with back‐transformed values in parentheses), with 95% confidence intervals (CI) computed using Dunnett's method. Numerator df for within collection comparisons: Gediflux 62, Watkins 274; ddf = denominator df

Species	Response	Collection	Predicted Mean	Predicted Difference With Solstice	95% CI for Predicted Difference With Solstice	Within Collection Comparisons		
						*F*	ddf	*P*
*R. padi*	No. nymphs	Gediflux	0.8811 (6.605)	−0.0417 (−0.091)	−0.0965 to 0.0132	1.48	826	0.012
		Watkins	0.9022 (6.984)	−0.0206 (−0.046)	−0.0493 to 0.0082	0.92	818	0.787
	Average weight of nymphs	Gediflux	−0.5616 (0.274)	0.0177 (1.042)	−0.0122 to 0.0476	3.55	812	<0.001
		Watkins	−0.5257 (0.298)	0.0536 (1.131)	0.0378 to 0.0694	1.64	810	<0.001
	Proportion nymphs surviving	Gediflux	–	–	–	1.12	819	0.258
		Watkins	–	–	–	0.93	819	0.743
*S. avenae*	No. nymphs	Gediflux	0.6630 (3.603)	−0.0284 (−0.063)	−0.0773 to 0.0204	1.04	714	0.399
		Watkins	0.6665 (3.639)	−0.0250 (−0.056)	−0.0512 to 0.0012	1.11	637	0.152
	Average weight of nymphs	Gediflux	−0.3949 (0.403)	−0.0106 (0.976)	−0.0488 to 0.0276	1.76	723	<0.001
		Watkins	−0.4033 (0.395)	−0.0189 (0.957)	−0.0386 to 0.0007	1.21	651	0.027
	Proportion nymphs surviving	Gediflux	–	–	–	1.49	705	0.011
		Watkins	–	–	–	0.93	705	0.753

### Wheat bulb fly field assessment

**Figure 4:**
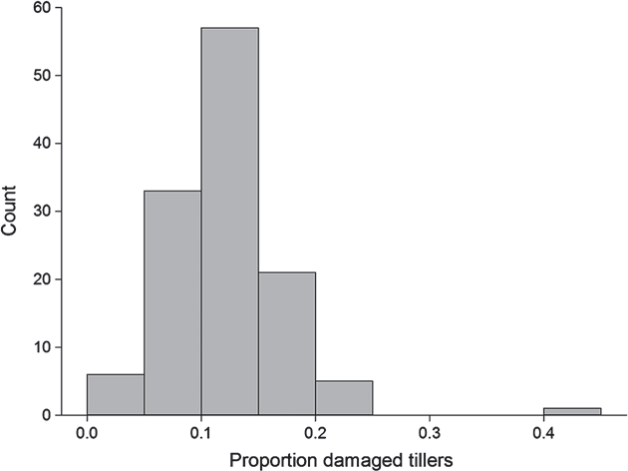
Histogram of the predicted proportion of damaged tillers for 122 wheat accessions from the Watkins collection and Paragon from the Gediflux collection, values are back‐transformed from the logit scale.

Despite a low wheat bulb fly infestation in 2013/2014, with damaged tillers per accession ranging from 2% to 42% (Fig. 4), the proportions of damaged tillers differed among accessions (*F*_122, 266_ = 1.33, *P* = 0.031). No difference was detected in number of larvae found inside the stems depending on accession (*F*_122, 266_ = 1.17, *P* = 0.145).

### Correlation between plant responses to wheat bulb fly and aphid species

Correlations were performed to establish whether there was an association between the performance of the different variables tested for the two aphid species and the proportion of tillers infested by wheat bulb fly on 122 accessions from the Watkins collection. The results showed that there was no correlation between infestation by wheat bulb fly larvae and performance of either aphid species, or in performance between the two aphid species. Number and weight of S. avenae nymphs were highly correlated (P < 0.001).

### Aphid field infestation

**Figure 5:**
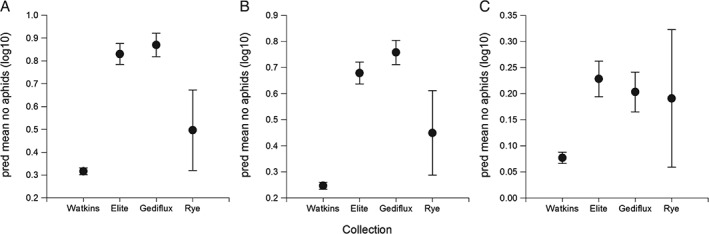
Mean number of aphids (log_10_ ± SEM) present on wheat from three different collections, Watkins, Gediflux and Elite lines, plus one Rye variety, in a field trial. (a) Total aphids, (b) Metopolophium dirhodum, and (c) Sitobion avenae.

The total count of aphids in the field was 4104, comprising 2580 M. dirhodum, 1479 S. avenae and 45 R. padi. Three outliers were removed from the analysis, these were unusually high counts (178, 190 and 380 compared with the fourth highest count of 53, N = 860) of S. avenae, one for each collection. Overall, mean total aphid field infestation pressure differed among collections (F_3, 682_ = 67.69, P < 0.001), with the lowest infestation occurring for the Watkins collection (Fig. 5a). However, mean total infestation differed among lines within the Watkins collection (F_143, 682_ = 1.51, P < 0.001), whereas no differences were found among the Elite varieties (F_13, 682_ = 1.54, P = 0.098), or among the lines of the Gediflux collection (F_11, 682_ = 0.76, P = 0.678). For M. dirhodum alone, which predominated in absolute aphid numbers, infestation pressure differed among the collections (F_3, 685_ = 63.90, P < 0.001; Fig. 5b), and while mean infestation did not differ among the Elite varieties (F_13, 685_ = 1.02, P = 0.426) or lines within the Gediflux collection (F_11, 685_ = 0.34, P = 0.975) there was some evidence for differences within the Watkins collection (F_143, 685_ = 1.23, P = 0.052). Sitobion avenae infested the collections in different numbers (F_3, 682_ = 6.96, P < 0.001). No differences were observed in numbers within the Watkins collection (F_143, 682_ = 0.82, P = 0.925) or the Gediflux collection (F_11, 682_ = 1.34, P = 0.199), but numbers of S. avenae differed among the Elite varieties (F_13, 682_ = 2.50, P = 0.002). There were not enough R. padi present for statistical analysis (range only from zero to six aphids in total per line).

## Discussion

This study, comprising a wide variety of wheat, including landraces from the Watkins collection deriving from before the green revolution, cultivars from the Gediflux collection (NW‐Europe, 1945–2000) and more modern UK Elite varieties, has not identified strong resistance to aphids or wheat bulb fly. Considerable variation was shown in insect performance among and within different wheat collections, with reduced susceptibility in a number of varieties. No association was identified between performance of the different insect species on individual varieties.

Identifying genetic diversity in diverse wheat collections is a prerequisite to creating a pre‐breeding germplasm to use for improvements in elite cultivars, including for pest resistance. Substantial loss of genetic diversity has been shown to have occurred from landraces to modern wheat cultivars (Reif *et al.*, 2005). Additionally, other work has shown there to be differences between the Watkins and Gediflux collections in most of the plant traits measured, such as flowering times, mature plant weight, thousand grain weight, grain surface area and width (Wingen *et al.*, 2014). We did not, however, see a difference in average performance of *R. padi* and *S. avenae* between the two collections, suggesting that during modern plant breeding, where improvements have focussed mainly on maximising yield, the plant effect on these aphid species has not been altered. In fact it could be inferred that a general loss of resistance traits happened before the rise of landraces, as resistance to aphid species has been reported in a number of diploid ancestors of wheat (Lamb & Migui, 2003; Migui & Lamb, 2004; Radchenko, 2011; Elek *et al.*, 2013).

This project has shown, however, that there is some variability in resilience to aphids and wheat bulb fly within the collections. For both aphid species, some lines showed moderate resistance in that aphid weight gain was comparatively reduced. For *R. padi* only, some lines from the Watkins collection supported an increased weight gain compared with the control, whereas only one line showed a reduction in nymph production. There was no overlap in the lines showing moderate resistance or increased weight gain for the different aphid species or variables in these lines, further highlighting the different responses of these two aphid species to the plants. Results from other screening studies searching for resistance to more than one insect pest are similarly variable. As in our study, an association mapping study on five major pests of wheat found considerable variation for resistance in hessian fly (*Mayetiola destructor* Say), Russian wheat aphid [*Diuraphis noxia* (Kurdjumov)], sunn pest (*Eurygaster integriceps* Puton), cereal leaf beetle (*Oulema melanopus* L.) and wheat stem saw fly (*Cephus cinctus* Norton), but none of the genotypes screened showed resistance to more than one pest (Joukhadar *et al.*, 2013). Other work, (Crespo‐Herrera *et al.*, 2013), found resistance to both *R. padi* and *S. avenae*, but focussed on germplasm derived from translocations with rye (*Secale cereale* L.) and the diploid wild wheat relative *Aegilops speltoides* Tausch, where they found lines showing resistance to both aphid species when tested at the seedling stage. These lines carried the complete 1R chromosome and the 1RS chromosome arm derived from E12165 wheat and Presto triticale (×*Triticosecale*), but the genetic basis for this resistance is not known. Another association mapping study comparatively analysed loci for resistance to *S. avenae* and *D. noxia* concluding that three loci identified could potentially control the response to both aphid species (Li & Peng, 2014).

The proportion of tillers infested by wheat bulb fly larvae differed among the 122 lines tested, with the highest infestation at 42% and the lowest at 2%. The larvae of the wheat bulb fly use chemotaxis and chemokinesis to locate host plants and are known to use primary and secondary metabolites, including carbon dioxide, hydroxamic acids, syringic acid and vanillic acid, as search cues. It has been suggested that primary metabolites such as carbon dioxide act as search triggers for wheat bulb fly larvae to induce searching behaviour which makes the larvae more likely to encounter the more plant specific secondary metabolites when they are near a suitable host plant (Rogers & Evans, 2013, 2014; Rogers *et al.*, 2013). Further studies are needed to assess whether exudates from attractive versus non‐attractive lines of the Watkins collection have different behavioural effects on wheat bulb fly larvae and if there are any differences in root exudate chemistry.

Field trials showed within collection differences in aphid performance, which will enable future studies on the chemistry and genetics involved in susceptibility differences to aphids. Fewer aphids populated lines from the Watkins collection overall under field conditions, which is in contrast with development data acquired in the laboratory bioassays where aphids were confined to the plant using a clip cage, and no difference was observed in aphid performance between the Watkins and Gediflux collections. This is interesting and suggests that there is a pre‐alighting cue deterring the aphid settlement, which could have important implications for aphid management in wheat production and should be studied further. It also demonstrates differences in aphid preference and performance on older plants in the field compared with seedlings in the laboratory, highlighting the need for phenotyping for aphid resistance at different plant growth stages (Migui & Lamb, 2004). This would be greatly improved by incorporating new technologies which would make field assessments less labour intensive.

In conclusion, the Watkins and Gediflux collections contain lines showing moderate resistance to the cereal aphids *R. padi* and *S. avenae*, as well as the wheat bulb fly *D. coarctata*, but no overlap was observed in resistance or susceptibility for these three insect species. Lack of correlation between behaviour of wheat bulb fly larvae in the field and the performance of two cereal aphid species *R. padi* and *S. avenae* in laboratory bioassays, further suggests that diverse traits are responsible for resistance to different insect species. Plants with resistance to multiple insects and pathogens would be highly desirable and further work will elucidate the potential for breeding in resistance to more than one species. Lines already demonstrating partial resistance will be investigated to identify further mechanisms towards a reduction in insect colonisation, feeding behaviour and performance.
